# Extensive bilateral diffuse infiltrates and deterioration of lung following infection with severe acute respiratory syndrome coronavirus 2 in a pregnant woman: a case report

**DOI:** 10.1186/s13256-021-03156-y

**Published:** 2021-12-13

**Authors:** Somayeh Moeindarbary, Salmeh Dadgar, Parvaneh Layegh, Zahra Shahriari, Faezeh Fayyaz, Sina Danesteh, Mahdi Rafiee, Milad Bahrami

**Affiliations:** 1 Department of Obstetrics and Gynecology, Neonatal and Maternal Research Center, Mashhad University of Medical, Mashhad, Iran; 2 Department of Radiology, School of Medicine, Mashhad University of Medical Sciences, Mashhad, Iran; 3 Student Research Committee, Faculty of Medicine, Mashhad University of Medical Sciences, Mashhad, Iran; 4 Student Research Committee, Faculty of Paramedical Sciences, Mashhad University of Medical Sciences, Mashhad, Iran

**Keywords:** Maternal death, SARS-CoV-2, Pulmonary fibrosis, Case report, COVID-19

## Abstract

**Introduction:**

Severe acute respiratory syndrome coronavirus 2 is the third member of the coronavirus family to cause global concern in the twenty-first century. Pregnant women are particularly at higher risk of developing severe viral pneumonia, possibly because of a partial immune suppression during their pregnancy. Under such critical and rapidly evolving circumstances, these poor findings might be helpful for the treatment of infected pregnant women with the 2019 novel coronavirus.

**Case presentation:**

In this study, we report the case of a 33-year-old Asian pregnant woman at 25 gestational weeks with coronavirus disease 2019 who developed severe complications, including hypoxemia, acute respiratory distress syndrome, pulmonary infiltration, and bilateral pleural effusion. She died 1 month after admission to the hospital.

**Conclusion:**

Pregnant populations are especially at higher risk of viral pneumonia development caused by severe acute respiratory syndrome coronavirus 2. Further research on the prevention and treatment of the new coronavirus is necessary.

## Introduction

Severe acute respiratory syndrome coronavirus 2 (SARS-CoV-2) is the novel coronavirus that was first discovered in the Chinese city Wuhan in December 2019 [1]. The outbreak of the SARS-CoV-2) was declared a global pandemic by the World Health Organization (WHO) on 11 March 2020. To date, there have been approximately 5 million confirmed cases, resulting in thousands of deaths worldwide [2]. The severity of symptoms ranges from mild symptoms to severe illness. The most frequent symptoms include cough, pharyngitis, fever, myalgia, chills, and dyspnea [3].

Severe dyspnea is caused by the progression of lung lesions in multiple lung lobes, and in some cases in advanced stages, white lung syndrome is observed. Lung lesions are manifested as airspace opacities on plain chest radiography, or ground-glass opacities or consolidation on chest radiography images, usually with a rounded morphology and a peripheral lung distribution, which is a diagnostic method for coronavirus disease 2019 (COVID-19) [4–6]. Pregnant women are particularly at higher risk of developing severe viral pneumonia, possibly because of a partial immune suppression during their pregnancy [7]. Pneumonia was reported as the third most common cause of mortality among pregnant women [8]. Previous data on Middle East respiratory syndrome (MERS) and severe acute respiratory syndrome (SARS) suggest that adverse pregnancy outcomes may occur due to the infection [9]. According to some studies, COVID-19 in the pregnant population can cause premature delivery and intrauterine growth restriction [6].

The effect of COVID-19 on pregnancy outcomes is not clear yet. Moreover, there has been no consensus on the maternal–fetal transmission of SARS-CoV-2 to date. Also, it has not been well understood whether preterm delivery ameliorates the symptoms of a critically ill mother. In this case report, we present the case of a 33-year-old Asian pregnant woman who was infected with SARS-CoV-2 in the late second trimester of pregnancy. Under such critical and rapidly evolving circumstances, these poor findings might be helpful for the treatment of pregnant women who are infected with SARS-CoV-2 in Iran and elsewhere.

## Case presentation

**Fig. 1:**
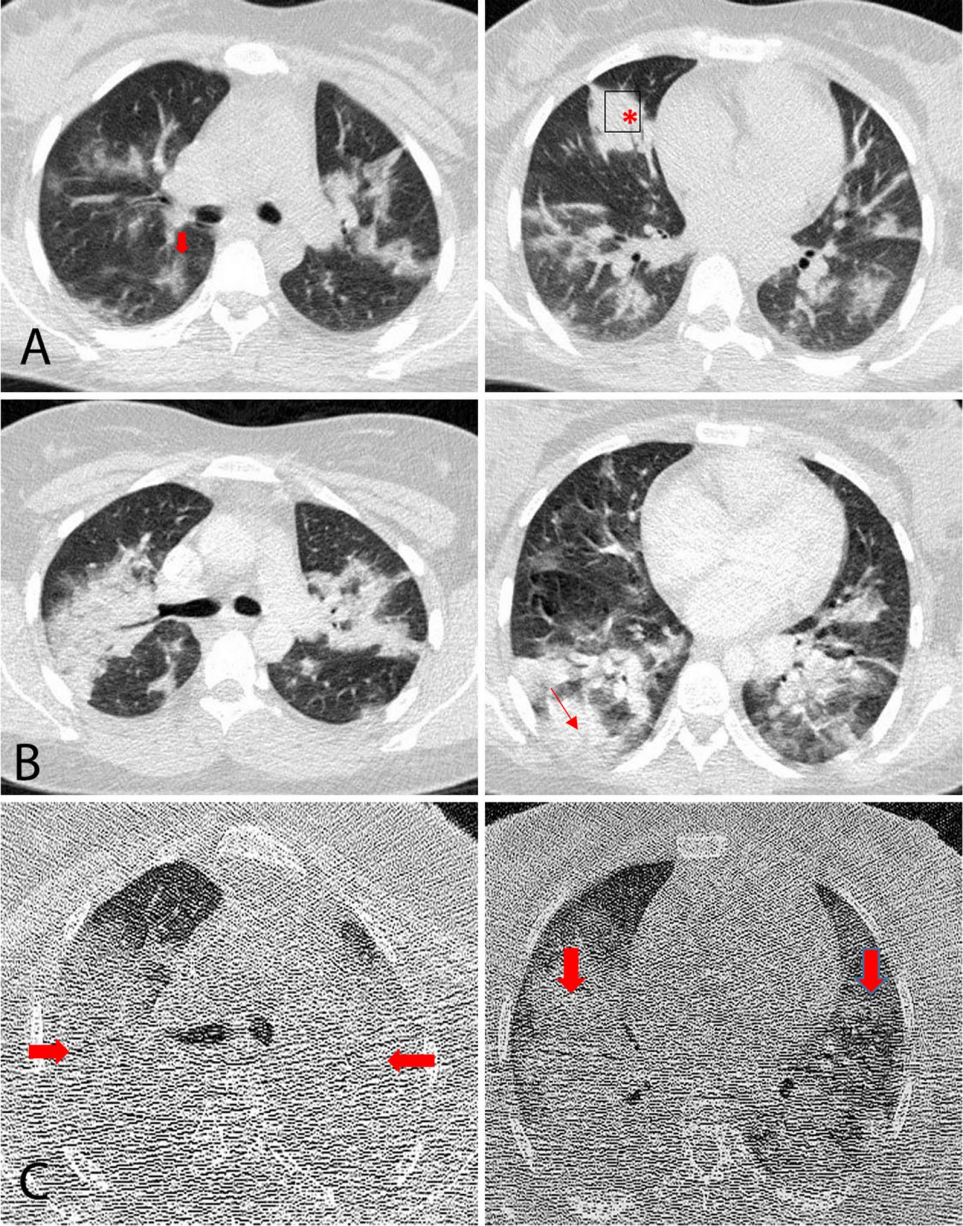
**A** Axial CT image from day 1 of hospitalization showing multifocal ground-glass opacities (  ) and nodular consolidation (*) in both lungs. **B** Newly developed opacities and decreasing density of the nodular opacities (  ) detected on follow-up CT on day 2 of hospitalization. **C** Last follow-up CT image on day 8, showing extensive bilateral diffuse infiltrates (  ) and deterioration of lung involvement

A 33-year-old Asian pregnant woman para 2, gravida 3, gestational age of 25 weeks, was hospitalized with fever, chills, shortness of breath, and myalgia. She had her first baby delivered by vaginal delivery 5 years ago and her second by cesarean section 2 years ago. The patient had no underlying diseases and did not have direct contact with COVID-19 cases. Vital signs of the patient were recorded as follows: respiratory rate (RR) 26 breaths per minute, blood pressure (BP) 110/70 mmHg, temperature (T) 39.5 °C, heart rate (HR) 110 beats per minute, and oxygen saturation (SpO_2_) 94%. The laboratory results showed that lymphocyte count was lower than normal (lymphocyte count 1 × 10^9^/L). While platelet count, hepatic enzymes, and creatinine levels were within the normal range, C-reactive protein level (CRP) was significantly increased. Coagulation function and blood biochemistry were normal (Table1). A GeneXpert SARS-CoV-2 RNA polymerase chain reaction (PCR) test was performed, and the result was positive. The patient had no complaints about uterine contractions, hemorrhage, and symptoms of amniorrhexis. Bilateral involvement was detected on chest computed tomography (CT), and on lung auscultation, diminished breath sound was detected. A day after the hospitalization, the patient was transferred to the intensive care unit (ICU) because of the shortness of breath (RR 32 breaths per minute) and low oxygen saturation (SpO_2_ 88%). CT chest on the second day of hospitalization revealed an exacerbation of pulmonary involvement. The patient was intubated 1 week after hospitalization owing to a reduction in the oxygen saturation to 80% and exacerbation of respiratory distress. Chest CT on the eighth day of hospitalization showed extensive bilateral diffuse infiltrates and deterioration of lung involvement (Fig. 1). From the beginning of hospitalization, despite the use of broad-spectrum antibiotic coverage, the patient’s fever was intermittent.

**Table 1:** Laboratory characteristics by the day of hospitalization

	Day 1	Day 4	Day 8	Day 12	Day 16	Day 20	Day 24	Day 28
White blood cell count, ×10^9^/L	10.1	9.2	8.1	7.3	9.0	8.3	15.2	20.5
Lymphocyte count, ×10^9^/L	1.0	1.0	1.0	0.8	0.8	0.5	0.7	0.5
Hemoglobin, g/L	12.8	11	8.8	9.6	9.4	8.3	11	10.5
Platelet count, ×10^9^/L	310	345	250	210	158	140	144	126
C-reactive protein, mg/L	79	50						
Alanine aminotransferase, U/L	15		24		28			35
Aspartate aminotransferase, U/L	25		30		30			32
Creatinine, μmol/L	0.6	0.8	0.9	1	1	1.1	1.5	1.5
VBG								
pH	7.39	7.46	7.41	7.49	7.39	7.42	7.19	
PO_2_, mmHg	59.5	42	40	35.7	74.9	38	43.7	
PCO_2_, mmHg	74.6	41.8	42.3	46.7	48	43.4	68.8	
HCO_3_, mEq/L	45.1	30.1	30.5	31.5	29.5	28.2	26.2	
BE, μmol/L	17.5	6.7	8.1	12.5	4.6	3.1	43.4	
O_2_ Sat, %	90.4	80.5	82	80	94.9	72.8%	65.4

One week after transferring to the ICU and due to the patient’s deteriorating condition, plasmapheresis was performed twice with 10 units of fresh frozen plasma (FFP) for the patient, which did not improve her condition. Also, due to a decrease in hemoglobin (HB 8.1 g/dl), two units of packed cells were transfused twice. Three weeks after hospitalization, ultrasounds were performed several times to assess fetal growth, and a sharp decrease in the amniotic fluid (amniotic fluid index 2.5 cm) and fetal growth retardation were observed. To rule out the possibility of a preterm ruptured membrane, an AmniSure test was performed twice, which was negative both times. Due to the condition of the fetus and the exacerbation of the patient’s respiratory condition, it was decided in the committee constituting of specialists in the special care and departments of lung and perinatology that the pregnancy should be terminated to improve the patient’s respiratory condition. Therefore, to induce cervical ripening, an intracervical Foley catheter was placed and extra-amniotic saline infusion was performed. After receiving three doses of vaginal misoprostol at doses of 50, 100, and 200 μg every 6 hours, a 600 g baby was born with a 1-minute Apgar score of 2 and a 5-minute Apgar score of 4, and the fetal heart rate was 145 beats per minute. The newborn was intubated and transferred to the neonatal intensive care unit (NICU) and died after 12 hours.

One week after the termination of pregnancy, due to the observation of bilateral pleural effusion on the chest CT, a chest tube was installed on both sides. Since no improvement in lung condition was observed and complete destruction of lung tissue was detected, the patient died 1 month after hospitalization.

## Discussion and conclusion

Lessons learned from the previous viral outbreaks have proven the negative impacts of viral infection on maternal, neonatal, and fetal outcomes. For instance, the total mortality rate of 1918 influenza was 2.6%, while it was 37% among pregnant women [10]. It was shown that MERS or SARS infection during pregnancy correlated with a higher incidence of severe maternal and fetal complications, such as admission to the ICU, preterm delivery, maternal and fetal mortality, and intrauterine growth restriction [11]. According to a study conducted by Nan Yu and associates, the pregnancy outcomes of pregnant women with COVID-19 are considerably better than those of pregnant women with SARS [12].

As previously discussed, pregnant women are especially susceptible to the adverse complications of viral pneumonia. However, for COVID-19, data regarding the differences in the clinical features of pregnant and nonpregnant women are yet limited. Chen *et al.* have reported that the clinical signs and symptoms of pregnant women with COVID-19 are similar to those of nonpregnant women [13]. Also, in a large case series conducted in the Hubei Province of China, the incidence of severe pneumonia in the pregnant population was the same as in the general population [14]. More clinical evidence is needed to support this relationship.

Fever is the most prevalent symptom in pregnant women infected with SARS-CoV-2, observed in 78% of the cases [15]. Consistent with previous studies, our patient presented with fever, chills, dyspnea, and myalgia. It is confirmed that COVID-19 is associated with a decrease in lymphocytes, as observed in our study [13]. One study performed chest CT scans on 15 pregnant women with COVID-19 and indicated that the most common early finding was ground-glass opacities, as seen in our case [15]. Many studies have reported that adverse outcomes and mortality are more likely to occur in patients with underlying medical conditions, whereas the present case did not have any underlying medical condition. In our study, the administration of convalescent plasma did not improve the clinical status of the patient, similar to the study of Niveditha that demonstrated that convalescent plasma has few or no clinical benefits for the treatment of COVID-19 [16]. It is not clear yet whether convalescent plasma with specific antibodies against SARS-CoV-2 that is collected from patients recovered from COVID-19 can improve the survival of critically ill patients. However, the plasma therapy has not been approved yet for use by the United States Food and Drug Administration, and numerous clinical trials are working on it [17].

Maternal complications of the present case were also in line with previous reports, which have found that pregnant women with severe or critical COVID-19 have a higher chance of developing respiratory failure, using mechanical ventilation, and maternal death, as well as fetal complications including preterm birth, intrauterine growth restriction, and intrauterine fetal death [18–21]. On the contrary, Zaigham *et al.* conducted a systematic review and, surprisingly, have suggested a lower rate of admission to the ICU, no maternal deaths, and only one neonatal death and one intrauterine fetal death [22].

In this patient, due to lack of improvement in respiratory status and progressive course of pulmonary destruction, as well as lack of fetal growth within 3 weeks and decreased immunity, it was decided to terminate the pregnancy to improve the respiratory condition. This is consistent with the rising concern that Castro *et al.* reported regarding preterm delivery in pregnant women with COVID-19. In their study, they found that 41% of deliveries in pregnant women with COVID-19 occurred before 37 weeks of pregnancy [23].

The limitation of our study was that no real-time polymerase chain reaction test was conducted on the premature infant, so we do not have any information about vertical transmission.

Since it has been proven in numerous studies that the pregnant population is especially at higher risk of viral pneumonia caused by coronaviruses, further research on the prevention and treatment of the new coronavirus is necessary.

## Data Availability

The patient’s information and medical records used for the case report are available from the corresponding author upon request.

## Abbreviations

COVID-19: Coronavirus disease 2019
WHO: World Health Organization
ARDS: Acute respiratory distress syndrome
SARS-CoV-2: Severe acute respiratory syndrome coronavirus 2
RT-PCR: Real-time polymerase chain reaction
CT: Computed tomography
BP: Blood pressure
HR: Heart rate
GGO: Ground-glass opacity
T: Temperature
RR: Respiratory rate
CRP: C-reactive protein
ICU: Intensive care unit
SpO_2_: Oxygen saturation
FFP: Fresh frozen plasma
NICU: Neonatal intensive care unit
ABG: Arterial blood gas
BE: Base excess
HB: Hemoglobin

## References

[1] Zhu N, Zhang D, Wang W, Li X, Yang B, Song J, *et al.* A novel coronavirus from patients with pneumonia in China, 2019. N Engl J Med. 2020;382(8):727–33. 10.1056/NEJMoa2001017 PMC7092803 31978945

[2] World Health Organization. https://covid19.who.int/.

[3] Zhong K, Wang RX, Qian XD, Yu P, Zhu XY, Zhang Q, *et al.* Neuroprotective effects of saffron on the late cerebral ischemia injury through inhibiting astrogliosis and glial scar formation in rats. Biomed Pharmacother. 2020;126:110041. 10.1016/j.biopha.2020.110041 32113053

[4] Huang C, Wang Y, Li X, Ren L, Zhao J, Hu Y, *et al.* Clinical features of patients infected with 2019 novel coronavirus in Wuhan, China. Lancet. 2020;395(10223):497–506. 10.1016/S0140-6736(20)30183-5 PMC7159299 31986264

[5] Nie S, Han S, Ouyang H, Zhang Z. Coronavirus disease 2019-related dyspnea cases difficult to interpret using chest computed tomography. Respir Med. 2020;167:105951. 10.1016/j.rmed.2020.105951 PMC7195086 32421539

[6] Dashraath P, Wong JLJ, Lim MXK, Lim LM, Li S, Biswas A, *et al.* Coronavirus disease 2019 (COVID-19) pandemic and pregnancy. Am J Obstet Gynecol. 2020;222:521. 10.1016/j.ajog.2020.03.021 PMC7270569 32217113

[7] Kareva I. Immune suppression in pregnancy and cancer: parallels and insights. Transl Oncol. 2020;13(7):100759. 10.1016/j.tranon.2020.100759 PMC7191218 32353791

[8] Visscher HC, Visscher RD. Indirect obstetric deaths in the state of Michigan 1960–1968. Am J Obstet Gynecol. 1971;109(8):1187–96. 10.1016/0002-9378(71)90664-8 5554850

[9] Schwartz DA, Graham AL. Potential maternal and infant outcomes from (Wuhan) coronavirus 2019-nCoV infecting pregnant women: lessons from SARS, MERS, and other human coronavirus infections. Viruses. 2020;12(2):194. 10.3390/v12020194 PMC7077337 32050635

[10] Gottfredsson M. [The Spanish flu in Iceland 1918. Lessons in medicine and history]. Laeknabladid. 2008;94(11):737–45. 18974435

[11] Schwartz DA, Graham AL. Potential maternal and infant outcomes from coronavirus 2019-nCoV (SARS-CoV-2) infecting pregnant women: lessons from SARS, MERS, and other human coronavirus infections. Viruses. 2020;12(2):194. 10.3390/v12020194 PMC7077337 32050635

[12] Yu N, Li W, Kang Q, Xiong Z, Wang S, Lin X, *et al.* Clinical features and obstetric and neonatal outcomes of pregnant patients with COVID-19 in Wuhan, China: a retrospective, single-centre, descriptive study. Lancet Infect Dis. 2020;20(5):559–64. 10.1016/S1473-3099(20)30176-6 PMC7158904 32220284

[13] Chen H, Guo J, Wang C, Luo F, Yu X, Zhang W, *et al.* Clinical characteristics and intrauterine vertical transmission potential of COVID-19 infection in nine pregnant women: a retrospective review of medical records. Lancet. 2020;395(10226):809–15. 10.1016/S0140-6736(20)30360-3 PMC7159281 32151335

[14] Yan J, Guo J, Fan C, Juan J, Yu X, Li J, *et al.* Coronavirus disease 2019 in pregnant women: a report based on 116 cases. Am J Obstet Gynecol. 2020;223:111. 10.1016/j.ajog.2020.04.014 PMC7177142 32335053

[15] Liu D, Li L, Wu X, Zheng D, Wang J, Yang L, *et al.* Pregnancy and perinatal outcomes of women with coronavirus disease (COVID-19) pneumonia: a preliminary analysis. AJR Am J Roentgenol. 2020;215(1):127–32. 10.2214/AJR.20.23072 32186894

[16] Devasenapathy N, Ye Z, Loeb M, Fang F, Najafabadi BT, Xiao Y, *et al.* Efficacy and safety of convalescent plasma for severe COVID-19 based on evidence in other severe respiratory viral infections: a systematic review and meta-analysis. CMAJ. 2020;197:E745–55. 10.1503/cmaj.200642 PMC7828893 32444482

[17] Sadeghnia HR, Shaterzadeh H, Forouzanfar F, Hosseinzadeh H. Neuroprotective effect of safranal, an active ingredient of *Crocus sativus*, in a rat model of transient cerebral ischemia. Folia Neuropathol. 2017;55(3):206–13. 10.5114/fn.2017.70485 28984113

[18] Alzamora MC, Paredes T, Caceres D, Webb CM, Valdez LM, La Rosa M. Severe COVID-19 during pregnancy and possible vertical transmission. Am J Perinatol. 2020;37:861–5. 10.1055/s-0040-1710050 PMC7356080 32305046

[19] Baud D, Greub G, Favre G, Gengler C, Jaton K, Dubruc E, *et al.* Second-trimester miscarriage in a pregnant woman with SARS-CoV-2 infection. JAMA. 2020;323:2198. 10.1001/jama.2020.7233 PMC7193526 32352491

[20] Hantoushzadeh S, Shamshirsaz AA, Aleyasin A, Seferovic MD, Aski SK, Arian SE, *et al.* Maternal death due to COVID-19 disease. Am J Obstet Gynecol. 2020;223:109.e1. 10.1016/j.ajog.2020.04.030 PMC7187838 32360108

[21] Pierce-Williams RAM, Burd J, Felder L, Khoury R, Bernstein PS, Avila K, *et al.* Clinical course of severe and critical COVID-19 in hospitalized pregnancies: a US cohort study. Am J Obstet Gynecol MFM. 2020. 10.1016/j.ajogmf.2020.100134. PMC7205698 32391519

[22] Zaigham M, Andersson O. Maternal and perinatal outcomes with COVID-19: a systematic review of 108 pregnancies. Acta Obstet Gynecol Scand. 2020;99:823–9. 10.1111/aogs.13867 PMC7262097 32259279

[23] Castro P, Matos AP, Werner H, Lopes FP, Tonni G, Araujo JE. Covid-19 and pregnancy: an overview. Rev Bras Ginecol Obstet. 2020;42(7):420–6. 10.1055/s-0040-1713408 PMC10798357 32559801

